# Unilateral, Isolated, Paediatric Lightning-Induced Cataract: A Case Report

**DOI:** 10.1155/2011/724395

**Published:** 2011-08-24

**Authors:** G. J. Rogers, R. Grotte

**Affiliations:** Division of Ophthalmology, Red Cross War Memorial Children's Hospital and the University of Cape Town, Klipfontein Road, Rondebosch, Cape Town 7700, South Africa

## Abstract

A six-year-old girl presented with gradual loss of vision in the left eye a year after sustaining a lightning strike while in her home. Examination revealed healed burns to her cheek, left arm, and right leg and a dense left cataract. There was no evidence of other ocular sequelae, and her right eye was normal. Cataract surgery and lens implantation were performed on the left eye with good results. Isolated, unilateral, paediatric cataract due to lightning is discussed.

## 1. Introduction

Despite the frequency of thunderstorms, direct strike by lightning is rare in South Africa. Cataract is the commonest ocular consequence of lightning injury, and this case involves the youngest patient in the literature with a unilateral, isolated lightning-induced cataract.

## 2. Case Report

**Figure 1:**
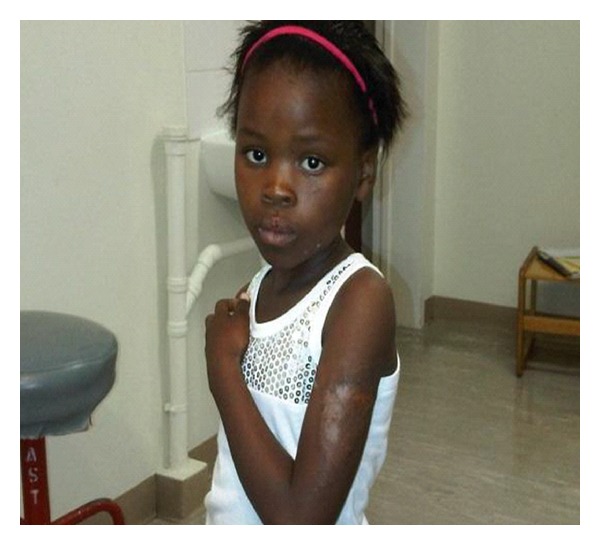
The subject demonstrating a lightning burn to her left arm.

A six-year-old girl from the Eastern Cape in South Africa presented with her mother to the Ophthalmology Unit at the Red Cross War Memorial Children's Hospital, with the complaint of painless, gradual loss of vision in her left eye (Figure 1). This symptom began in the months subsequent to a lightning strike to her, while at home in Ngcobo, which tragically also caused the sudden death of a younger sibling and cousin. She was referred to us by the school's optometry service. 

**Figure 2:**
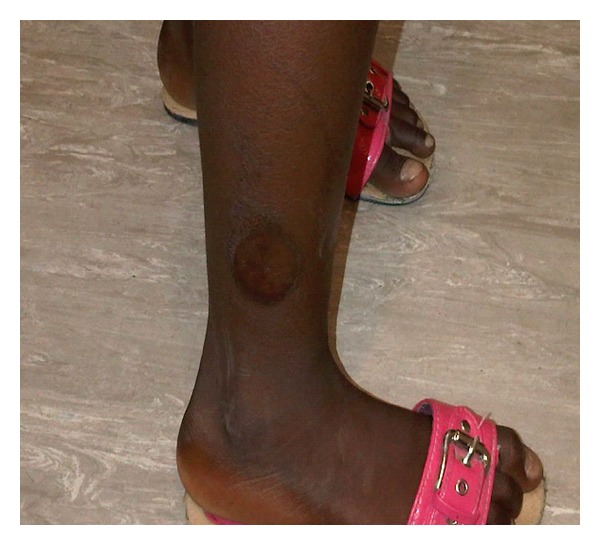
The exit wound on the right leg.

In the lightning incident, our patient sustained annular burns to the outer aspect of her right lower leg and left arm (Figures 1 and 2). Her mother described swelling across her daughter's face from right superior to left inferior, which healed without scarring. She had been admitted for the treatment of these burns for three weeks to the local All Saint's hospital. Ocular and systemic history was unremarkable. She had had a normal birth, at term, and had no prior history of serious illness, poor vision, ocular trauma, or red eye.

**Figure 3:**
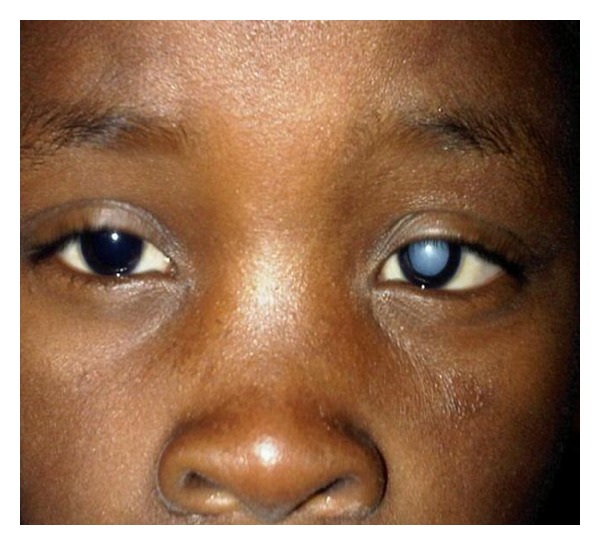
A dense left cataract.

General examination revealed scars to the skin of the right leg and left arm, as well as a small area of hypopigmentation on the cheek below her left eyelid (Figure 3). Otherwise, there were no systemic abnormalities. 

Right unaided visual acuity was 20/20 with Snellen's numbers, and the left eye perceived light projection in all quadrants. There were normal ductions and versions of both eyes and a mild left esotropia, although this was estimated with poor fixation. The pupillary reactions were normal and brisk. The ocular adnexa, conjunctiva, cornea, and iris were normal. There was no evidence of blunt trauma to the eyes. The intraocular pressure in both eyes was 12 mmHg. The left lens was densely cataractous precluding fundal visualisation (Figure 2) and had marked anterior subcapsular vacuoles. The examination of the right eye was normal, including a clear lens. A B-scan of the left eye was normal. Potential acuity testing was not performed, as we have found it unreliable in eyes with vision worse than 20/200. TORCH serology screen was negative.

Arrangement was made for lens washout and intra-ocular lens implantation on the next available elective list. The surgery was uneventful, and a 28D intraocular lens (MA60-AC, Alcon Laboratories, Inc., Tex, USA) was implanted in the capsular bag. 

On the first postoperative day, the vision was 20/20 without improvement despite preexisting corneal cylinder. The intraocular lens was well-centered in the bag and the cornea clear. Fundus examination was normal, with no posterior vitreous detachment, retinal scars, or embryological abnormalities. OCT done at one month showed no macular anatomical abnormalities. There was no residual esotropia, and the patient showed no evidence of amblyopia. Stereoacuity testing with the Stereo Fly test, as well as the Worth 4-dot testing, was also normal.

## 3. Discussion

Lightning kills up to ten inhabitants of certain population-dense parts of South Africa, annually [1]. The Highveld, for example, has a mortality rate of 6.3 deaths per million, significantly higher than the worldwide average of 1.7. From US data, one may extrapolate that in the same area, ten times more survived lightning [2].

Lightning has been known to cause cataract for almost three hundred years. Unfortunately, there have been no conclusive studies on the incidence of cataract amongst lightning victims. Industrial electric shock is associated with a 5–20% incidence [3]. Cataract is widely acknowledged as the commonest of ocular sequelae. 

It is rare for a child to survive lightning and rarer to develop isolated, unilateral cataract. It is,however, usual, as in this child, to have the more advanced cataractogenesis ipsilateral to the side of the shock; a shock seemingly targeting the orbit [4]. In the UK, over half of those struck are also struck indoors [5]. Cataractogenesis has been recognized to have occurred immediately but more commonly, as in our patient, to have had a latent period of months to years. Direct lightning strikes can expose victims to up to 30 kiloamperes of current or 50 to 100 million volts. In a major review, Norman and coworkers discuss in detail the pathophysiology of lightning damage [2]. The lens proteins, capsule, and epithelial cells are susceptible to a combination of mechanical shock, heat, or sudden vasoconstriction. Hanna and Fraunfelder have emphasised the role of heat from resistance through the pigmented part of the iris, which damages lens proteins indirectly [5]. This heat is presumably most active at the lens surface, resulting in the typical anterior and posterior subcapsular lightning cataract, often progressing to mature cataract. This is distinct from the isolated anterior subcapsular opacification from industrial electric shock.

There are several case reports of lightning cataract in the literature. Grewal et al. described a dense cataract in one eye and the early, subclinical anterior subcapsular opacities developing in the other eye in a 22-year-old man after suffering an electric shock [6]. These subclinical changes were only detectable by Pentacam's imaging. This and other studies identify the asymmetry of cataractogenesis in these patients. 

Gupta et al. describe a case of a 30-year-old man with bilateral cataract with posterior vitreous detachments induced by lightning [7]. In this paper, it is suggested that the site and severity of exit and entry wound are not related or proportional to other injuries. 

Espaillat et al. reported a case of a 30-year old sustaining bilateral cataract, posterior vitreous detachments, macular holes, and an inferotemporal retinal detachment from lightning [8]. 

To our knowledge, there are only two paediatric cases in the literature involving older children. Hanna and Fraunfelder in Injury [5] discuss a 9-year-old boy who was struck by lightning transmitted through a telephone. A year afterwards he had developed a typical unilateral, posterior subcapsular cataract reducing his vision to 6/60 in his right eye. 

A 13-year-old boy suffered a strike to his temple while hiking, and the consequent maculopathy left him with 20/25 and 20/60 vision [9]. 

Despite the strong history and physical evidence, all efforts were made to exclude other commoner causes of unilateral paediatric cataract. It was appropriate to exclude congenital infection and to evaluate the posterior segment with ultrasound and, later, coherence tomography. This was both for causes of congenital cataract as well as for lightning-induced ocular sequelae which may have altered management or prognosis.
